# Multiomics provides insights into dynamic changes of aromatic profile during flue-curing process in tobacco (*Nicotiana tabacum* L.) leaves

**DOI:** 10.1186/s12870-025-06273-8

**Published:** 2025-02-24

**Authors:** Ruiqi Wang, Binghui Zhang, Gang Gu, Jianfeng Lin, Wenwei Zhang, Dongwang He, Fei Wang, Liao Jin, Xiaofang Xie

**Affiliations:** 1 College of Life Sciences, Fujian Agriculture & Forestry University, Fuzhou, 350002 China; 2 Institute of Tobacco Science, Fujian Provincial Tobacco Company, Fuzhou, 350003 China; 3 Yanping Branch of Nanping Tobacco Company, Nanping, 353000 China; 4 Jianning Branch of Sanming Tobacco Company, Sanming, 354500 China; 5 Fujian Key Laboratory of Crop Breeding by Design, Fujian Agriculture & Forestry University, Fuzhou, 350002 China

**Keywords:** Metabolomics, Tobacco (*Nicotiana tabacum* L.), Aroma, Enzyme activity, Volatile compounds

## Abstract

Tobacco (*Nicotiana tabacum* L.) is a globally crop due to its distinctive flavor and economic value. In this study, we systematically analyzed the dynamic changes in volatile substances, broad-spectrum metabolites, enzymes, and biochemical compounds in tobacco leaves during flue-curing process. Combining metabolomics with enzyme activity and biochemical analysis, we identified that 43℃ is a critical period for enzyme activity and metabolite transitions, while 45 ℃ requires stringent moisture control. During the T3 stages, phenolic acids, amino acids, and derivatives were notably enriched, with increases of 19.58-fold, 18.59-fold, and 17.33-fold in lmmn001643, MWS20633g, and Lmhn004756, respectively. These compounds may serve as candidate biomarkers for non-volatile compounds. Aroma dynamics primarily contributed to the green and sweet flavor of flue-cured tobacco leaves, and the key aroma components included D114, KMW1317, and KMW0466. Differential volatile and non-volatile metabolites were enriched in four pathways, including monoterpenoid biosynthesis, tyrosine metabolism, phenylalanine metabolism, and phenylpropanoid biosynthesis. These pathways are closely related to phenylalanine ammonia-lyase and the synthesis of chlorogenic acid and rutin, which influence the aroma quality, aroma intensity, irritation, and volatility of tobacco. Additionally, the contents of caffeic acid, ferulic acid, sinapic acid, and PAL activity in phenylpropanoid biosynthesis pathway, increased with the rising temperature, accelerating reactions with alcohols and leading to increase lignin formation. This study enhances our understanding of the dynamic changes in the aroma and metabolic substances of Cuibi 1(CB-1) at the critical stages of the curing process and offers valuable insights for process improvement.

**Supplementary Information:**

The online version contains supplementary material available at 10.1186/s12870-025-06273-8.

## Background

Tobacco (*Nicotiana tabacum* L.) is an important crop with substantial economic value worldwide, and its aroma is one of its most appealing characteristics [1]. The curing process is a crucial stage in the production of tobacco, during which the leaves undergo significant changes in color, aroma and substance. This transformation is driven by a combination of natural chemical processes and human intervention [2], resulting in the development of the tobacco’s desirable characteristics. Understanding these changes is essential for producing high-quality tobacco products, such as cigarettes, and pipe tobacco [3].

The flue-curing process involves a series of meticulously controlled steps, each playing a distinct role in the alteration of the tobacco’s color and substance [4]. As the temperature increases, tobacco leaves gradually turn yellow. Comparative proteomics analysis revealed that plastid pigment metabolism contributes to these color changes during curing [1]. Additional, significant browning reactions occur when the temperature reaches 46℃, the point at which cell death typically happens in most plants [5]. Browning reaction is one of the key factors affecting the economic value of tobacco. Furthermore, factors such as residual ash on leaves, pungent odor, and insufficient aroma also impact the final quality of the tobacco [6, 7]. Enzymes play a crucial role in this dynamic process. Starch degradation, in particular, is vital for achieving a desirable postharvest cigarette by softening and sweetening the aroma. It is essential to explore changes in enzyme activity and biochemical substances during tobacco curing [8].

The composition and content of flue-cured tobacco aroma substances directly determine the quality of cigarette [9]. Volatile metabolites and metabolizing enzymes [10] undergo a series of oxidation, condensation, degradation, and polymerization reactions during this process, ultimately imparting the special flavor of tobacco [2]. After the degradation of aromatic amino acids in tobacco leaves, benzaldehyde, benzyl alcohol, phenylacetaldehyde and phenethyl alcohol are mainly formed into compounds with small molecular weight and strong volatility, which have floral, almond, nutty and burnt aromas, respectively, and contribute greatly to the fruity aroma and fragrance of flue-cured tobacco [11]. In recent years, volatile metabolomics has been frequently employed to investigate the alterations of aroma compounds, such as the compositional changes of aroma substances in plants like tea leaves [12] and kiwifruits [13]. Nevertheless, the aroma compounds that play a pivotal role during tobacco curing remain unexplored.

Improving tobacco quality through artificial flue-curing control requires a comprehensive understanding of the role of metabolites and the mechanisms involved in the formation of flavor-active compounds. However, research on the changes in aroma precursor content and their formation mechanisms in tobacco leaves during the curing process is still limited. Performing qualitative and quantitative analyses of metabolome and biochemical substances is essential for elucidating the interactions between enzymes, substrates, and intermediates in the metabolic pathways during curing. This approach will help to explore overall metabolic changes and reveal the metabolic basis for tobacco quality formation. In this study, we employed multi-omic and various physiological and biochemical methods to comprehensively analyze the metabolites, volatile substances, polyphenols, enzymes, and other biochemical substances to explore the mechanisms underlying quality formation during the critical stages of tobacco curing process. The findings will provide valuable insights for optimizing the curing process.

## Methods

### Plant materials and sample Preparation

**Fig. 1:**
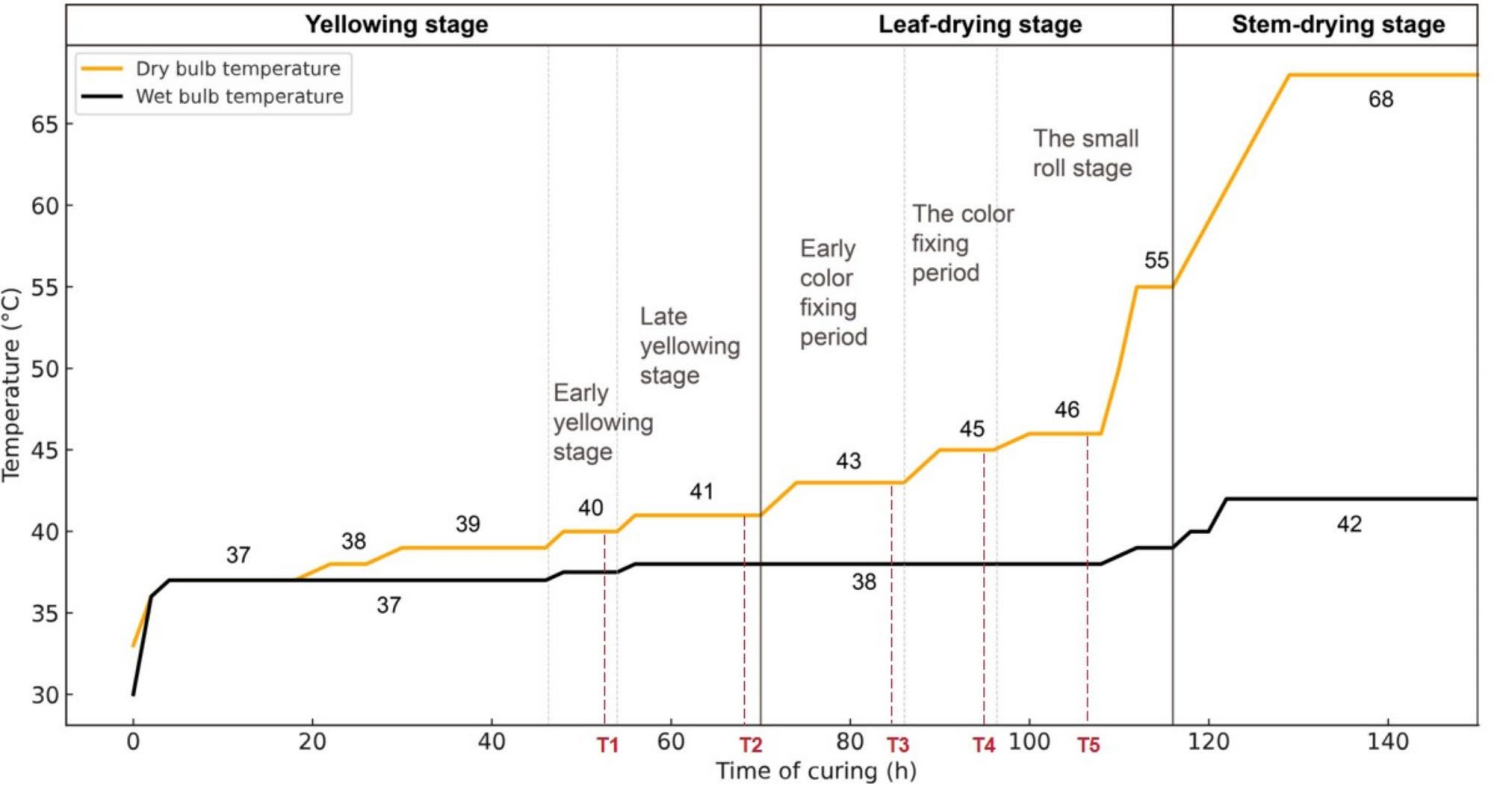
Dry- bulb and wet-bulb temperature in flue-curing stages

The upper leaves (leaves 13–15) of *Nicotiana tabacum* Cuibi 1 (CB-1), a variety renowned for its refined aroma, smooth flavor, and high industrial applicability, were sourced from a well- managed farmland in Jukou Township, Yanping District, Nanping City, Fujian Province, China, and served as the experimental materials [14]. Tobacco leaf samples were collected from six key stages during the curing process (Fig. 1), including the initial point (0 h), the early yellowing stage at 40 °C (52 h), and the late yellowing stage at 41 °C (68 h), as well as the early color fixing period at 43 °C (84 h), the color fixing period at 45 °C (94 h), and the small roll stage at 46 °C (106 h). These stages were recorded as T0, T1, T2, T3, T4 and T5, respectively. For each stage, six leaves were collected as a sample, with three biological replicates, totaling 18 samples. The front and back 1/4 parts of the leaves were cut off, and samples were taken by punching holes along both sides of the main vein. The samples were then wrapped in tin foil, labeled, placed in liquid nitrogen, and stored in a freezer at -80 °C for further analysis.

### Analysis of chemical composition and chlorophyll content

The chemical composition and chlorophyll content were analyzed for samples collected at six stages (T0 - T5). Total nitrogen, nicotine, starch, total soluble sugar, and reducing sugar content were assessed using the methods outlined in the Chinese tobacco industry standards (YC/T 161–2002, YC/T 160–2002, YC/T 216–2007, and YC/T 159–2002) through a continuous flow method. All chemical composition measuring methods followed the descriptions in the Chinese tobacco industry standard method [15]. Protein content was examined using the biuret method. The total nitrogen content of plant samples was determined using the automated nitrogen analyzer method, following the protocol outlined in NYT 2419 − 2013. Chlorophyll was extracted using 95% ethanol and quantified following the method described by He and Gan [16]. The supernatant was measured at 649 nm and 665 nm using a spectrophotometer (UV-1780, Shimadzu, Japan). Chlorophyll contents were calculated, and mean values were obtained based on three biological replicates.

### Enzyme activity measurement

The enzyme activity were analyzed for samples collected at the five distinct stages (T1 - T5). Following the methods described in precious studies, the activities of phenylalanine ammonia-lyase (PAL) [17, 18], polyphenol oxidase (PPO) [19], peroxidase (POD) [20], and superoxide dismutase (SOD) [21, 22] at five stages during the curing process were measured. Approximately 0.2 g of each sample was weighed and examined using BoxBio’s assay kit (Beijing Boxbio Science and Technology Co., Ltd., Beijing, China). The samples were then assayed using an ultraviolet spectrophotometer according to the manufacturer’s instructions. Three biological replicates were set up for each sample.

### Volatile metabolites analysis of tobacco leaves during curing process

Volatile organic compounds (VOCs) were analyzed using headspace solid-phase microextraction (HS-SPME) and gas chromatography-mass spectrometry (GC-MS). Tobacco leaves from five stages (T1-T5) were ground into powder, and 500 mg (1 mL) of the powder was immediately transferred to a 20 mL headspace vial with a saturated NaCl solution to inhibit enzyme reactions. The samples were placed at 60 °C for 5 min, and a 120 μm DVB/CWR/PDMS fiber was exposed to the sample’s headspace at 60 °C for 15 min. VOCs were then desorbed from the fiber in the GC injection port at 250 °C for 5 min in splitless mode. Identification and quantification of VOCs were conducted using an Agilent 8890 GC and 7000D mass spectrometer with a DB-5MS capillary column. Helium was used as the carrier gas at 1.2 mL/min, and the injector temperature was 250 °C. The oven temperature was programmed from 40 °C to 280 °C, with a final hold of 5 min. Mass spectra were recorded in electron impact (EI) ionization mode at 70 eV, with SIM mode used for analysis [23].

### None-volatile metabolites analysis of FTLs during curing process using wide-target metabolomics

Flue-cured of tobacco leaves (FTLs) (T1-T5) were freeze-dried using a vacuum freeze-dryer (Scientz-100 F). The freeze-dried FTLs were then crushed using a mixer mill (MM 400, Retsch) with a zirconia bead for 1.5 min at 30 Hz. A 50 mg portion of the lyophilized powder was dissolved in 1.2 mL of 70% methanol solution, followed by vortexing for 30 s every 30 min, repeated six times in total. The extracts were then centrifuged at 12,000 rpm for 3 min, and the supernatant was filtered (SCAA-104, 0.22 μm pore size; ANPEL, Shanghai, China) before UPLC-MS/MS analysis. The FTL extracts were analyzed using a UPLC-ESI-MS/MS system (UPLC, ExionLC AD; MS, Applied Biosystems 6500 Q TRAP). The analytical conditions were as follows: UPLC column: Agilent SB-C18 (1.8 μm, 2.1 mm × 100 mm); flow rate: 0.35 mL per minute; column oven temperature: 40 °C; injection volume: 2 µL. The effluent was alternatively connected to an ESI-triple quadrupole-linear ion trap (QTRAP)-MS.

### Statistical analysis

Primary metabolites were annotated using the self-built human metabolome database (MWDB) (Wuhan Metware Biotechnology Co., Ltd.; https://www.metware.cn, Wuhan, China). Unsupervised principal component analysis (PCA) was performed using the statistics function within R (www.r-project.org). The data were unit variance scaled before performing unsupervised PCA. The thresholds for significantly regulated metabolites were set at|Log2FC| ≥ 1.0, VIP ≥ 1, and *P* < 0.05. VIP values were extracted from the orthogonal partial least-squares discriminant analysis (OPLS-DA) results, which also included score plots and permutation plots generated using the R package MetaboAnalystR. To avoid overfitting, a permutation test with 200 permutations was performed. Identified metabolites were annotated using the KEGG Compound database (http://www.kegg.jp/kegg/compound/), and annotated metabolites were then mapped to the KEGG Pathway database (http://www.kegg.jp/kegg/pathway.html). Pathways with significantly regulated metabolites were further analyzed using metabolite set enrichment analysis (MSEA), with their significance determined by hypergeometric test p-values. In volatile metabolites analyzes, relative odor activity value (rOAV) is a method for determining key flavor compounds in food based on the sensory threshold established by the binding compounds. $\:{\text{r}\text{O}\text{A}\text{V}}_{\text{i}}=\frac{{\text{C}}_{\text{i}}}{{\text{T}}_{\text{i}}}$ where rOAVi is the relative odor activity value of compounds, Ci followed by the relative content of the compound (ug/g or ug/ml); Ti is the threshold of the compound (ug/g or ug/ml) [24, 25].

## Results

### Dynamic changes of morphological characteristics and chemical composition content of FTLs during curing process

**Fig. 2:**
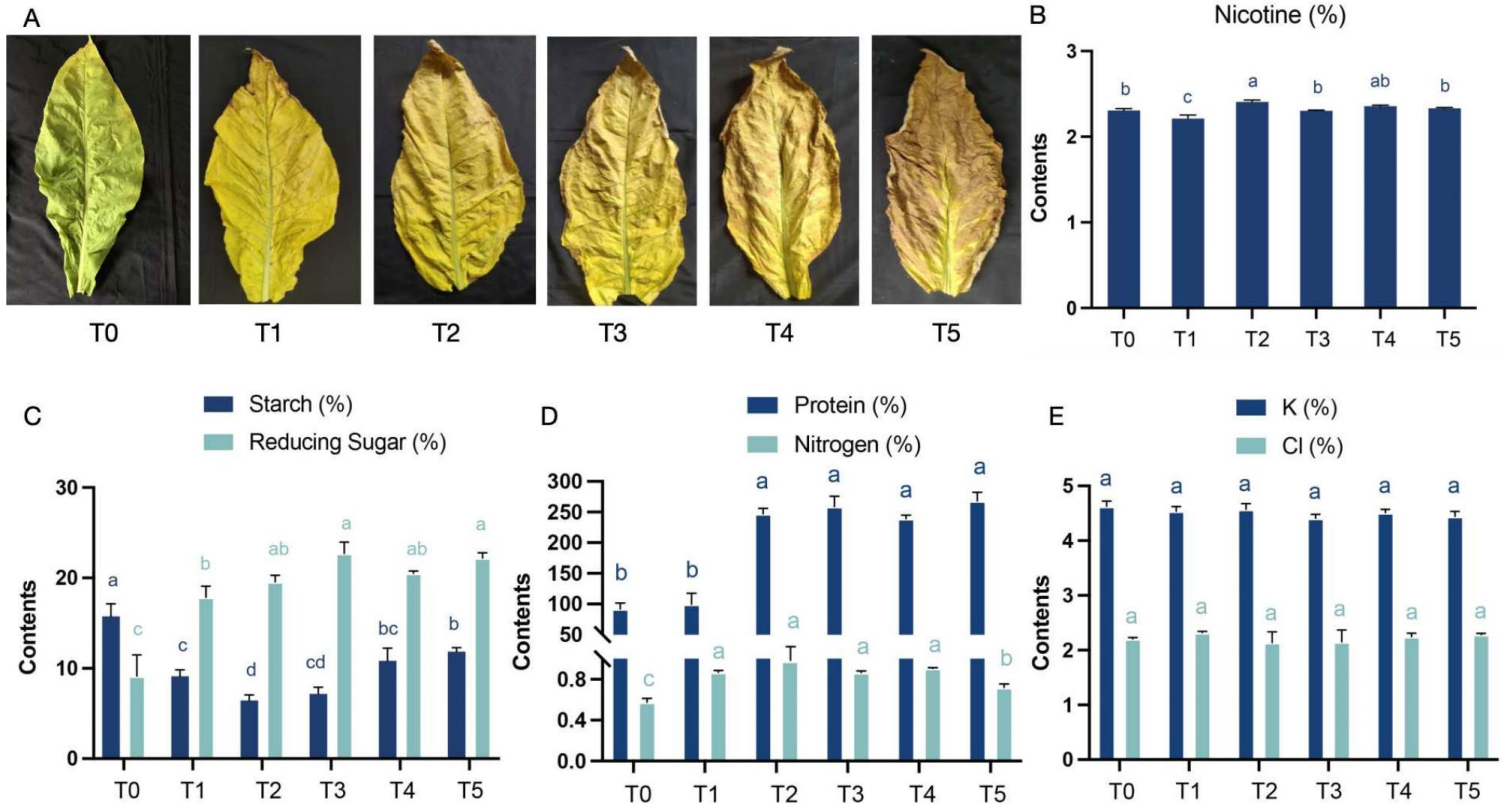
Dynamic changes of leaf characteristics at six stages of curing process. The change of appearance (**A**), nicotine content (**B**), starch and reducing sugar (**C**), protein and nitrogen (**D**) and potassium and chlorine (**E**) in tobacco leaves under different curing stages. Error bars indicate the means ± SD (*n* = 3). Lowercase letters represent significant differences for different stages during curing process (*p* < 0.05)

As curing progresses from T0 to T5, the yellowing rates of flue-cured tobacco leaves increased gradually, turning yellowish-brown color with rising temperature. Concurrently, the moisture content and flatness of the leaves gradually decreased, making them more flexible (Fig. 2A). The decrease in chlorophyll content with the increasing of temperature was consistent with the observed yellowing (Additional file 1: Fig S1). The reducing sugar content, serving as a source of umami compounds, exhibited an increasing trend from T0 to T3 (Fig. 2C).This rise significantly reduced the stimulating qualities while enhancing the fresh and brisk character of FTLs [26, 27]. Starch content showed a downward trend from T0 to T3, likely converting into sucrose and reducing sugars, followed by an increase as temperatures continued to rise (Fig. 2C). This trend can likely be attributed to the intensification of browning reactions in the leaves, which is accompanied by cell vacuole rupture and the loss of water [5]. Protein levels increased significantly at the T2 stage, while nitrogen content rose from T1 to T2 but subsequently declined at T5 (Fig. 2D). During the mid-to-late curing stages, the elevated temperatures may lead to the decomposition of nitrogenous compounds, forming volatile nitrogen-containing substances (e.g., ammonia) or non-volatile nitrogen products. These volatile compounds are released into the environment, resulting in a progressive reduction in nitrogen content. The changes in chlorine and potassium content were negligible throughout the curing process (Fig. 2E).

### Dynamic changes in enzyme activity of FTLs at five curing stages

**Fig. 3:**
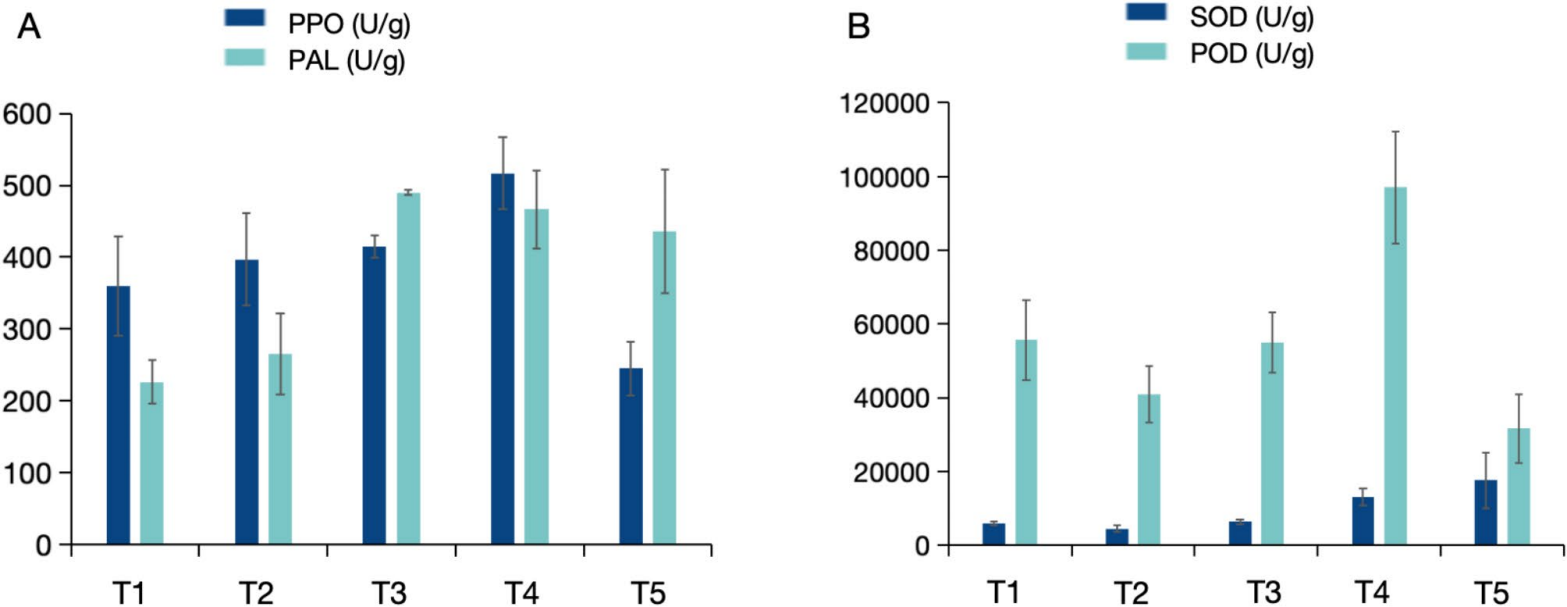
The dynamic changes of enzymatic activity in tobacco leaves under five stages of the curing process. (**A**) PPO and PAL activity, (**B**) SOD and POD activity

To investigate the changes in intrinsic substances associated with quality formation during the curing processes of FTLs, we measured the activities of several crucial enzymes. These included superoxide dismutase (SOD), which plays an important role in biological antioxidant systems; polyphenol oxidase (PPO) and peroxidase (POD), both of which are involved in browning reactions; and phenylalanine ammonia-lyase (PAL), a key and limiting enzyme in the phenylpropanoid metabolic pathway. As the temperature increased, the activity of SOD rose gradually from 5967.23 U/g to 17579.97 U/g (Fig. 3B). In contrast, PPO activity initially increased, peaking at T4 stage with a value of 759.47 U/g and then decreases to 244.95 U/g at T5 stage (Fig. 3A). The POD activity also peaked at T4 stage, reaching 96996.77 U/g, while PAL activity peaked at T3 stage with a value of 490.02 U/g and then gradually decreased with the rising temperatures (Fig. 3).

### Dynamic changes in volatile metabolites of FTLs during curing process

**Fig. 4:**
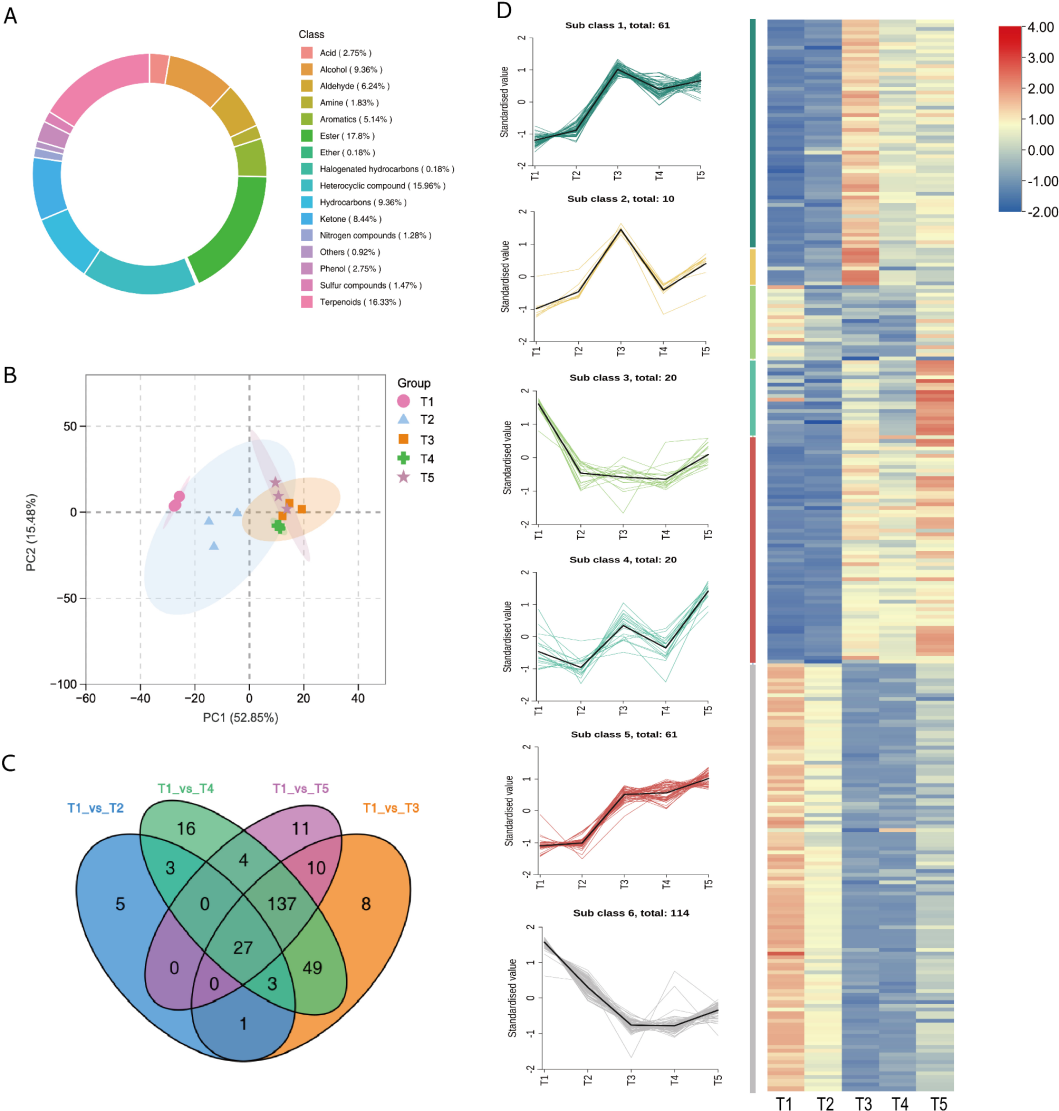
Identification of volatiles profiling in FTLs. (**A**) Percentages of different types of volatiles; (**B**) Principal component analysis of volatiles identified from the 15 samples; (**C**) Venn diagram showing the numbers of common and specific differential volatiles from different comparison; (**D**) Line charts plot for K-means clustering analysis of differential volatiles and visualized in clustering heatmap

A total of 545 volatile organic compounds were identified in T1 ~ T5 curing stages using HS-SPME GC/MS. These VOCs (Volatile organic compounds) were categorized into 16 categories (Fig. 4A). Among them, ester were the most prevalent, accounting for 17.8%, followed by terpenoids (16.33%), heterocyclic compounds (15.96%), and alcohol (9.36%). These categories were the main VOCs identified in FTLs. Esters, terpenoids, ketones, and alcohols contribute to a wide range of aromas.

Principal component analysis (PCA) of the 15 samples showed that the analysis method was stable and the data quality was suitable for further analysis (Fig. 4B). The PCA plots for these samples revealed that the component 1 (PC1) accounted for 53.11% of the variance, while component 2 (PC2) explained 16.28%. The distribution of sample points within each treatment group was relatively concentrated, indicating that the samples in each group had good repeatability and consistent metabolites profiles.

To investigate the dynamic changes in VOCs during the curing process, differentially volatiles at five curing stages (T1 ~ T5) were identified. Four comparisons were analyzed, namely, C1 (T1 vs. T2), C2 (T1 vs. T3), C3 (T1 vs. T4), C4 (T1 vs. T5). The result revealed that 39, 235, 239, and 189 differentially volatiles were identified in comparisons of C1, C2, C3 and C4 (Additional file 2: Table S1), respectively (Fig. 4C). Notably, the number of different volatiles increased substantially from 39 in C1 to 235 in C2, representing a more than six fold increase (Fig. 4C). This suggested a significant change in volatiles of FTLs from the early yellowing period (T1 stage) to the early stage of color fixing (T3 stage). After the color fixing period, the metabolic changes proceeded more slowly. Venn analysis (Fig. 4C) identified 27 common volatiles in the four comparisons, indicating that these substances undergo continuous changes during the curing process. The overlapping VOCs, with their individual aroma compounds, likely contribute to the varied flavors of cured tobacco.

To analyze the change trends of all the VOCs, the relative contents were standardized and centralized using K-means clustering analysis. A total of 286 significantly regulated VOCs were identified in four comparisons (C1-C4) and were clustered into six distinct profiles, which were visualized in a clustering heatmap (Fig. 4D). Approximately 53% of volatiles showed an upward trend during the curing stages. Among them, the content of 71 volatiles in subclasses 1 and 2 peaked at the color fixing stages (T3 stage) and then decreased at T4 stages. Conversely, the content of 134 volatiles in subclasses 3 and 6 decreased from stage T1 to T4 before gradually increasing in stage T5. Additionally, 61 volatiles in subclass 5 exhibited a continuous increase during the curing process. Notably, these included compounds with an rOAV > 1, such as KMW0608(3-Mercaptohexanol) with sulfurous, fruity, tropical aroma; NMW0016(Acetic acid, cyclohexyl ester) with fruity, sweet, musty, and ethereal aroma, WMW0050(2(4 H)-Benzofuranone, 5,6,7,7a-tetrahydro-4,4,7a-trimethyl-, (R)-)with musky, coumarin aromas, KMW0123(4-Heptenal, (Z)-) with oily, fatty, green, dairy, milky, and creamy aroma.

### Key aroma-active compounds forming the different characteristic aroma types

**Fig. 5:**
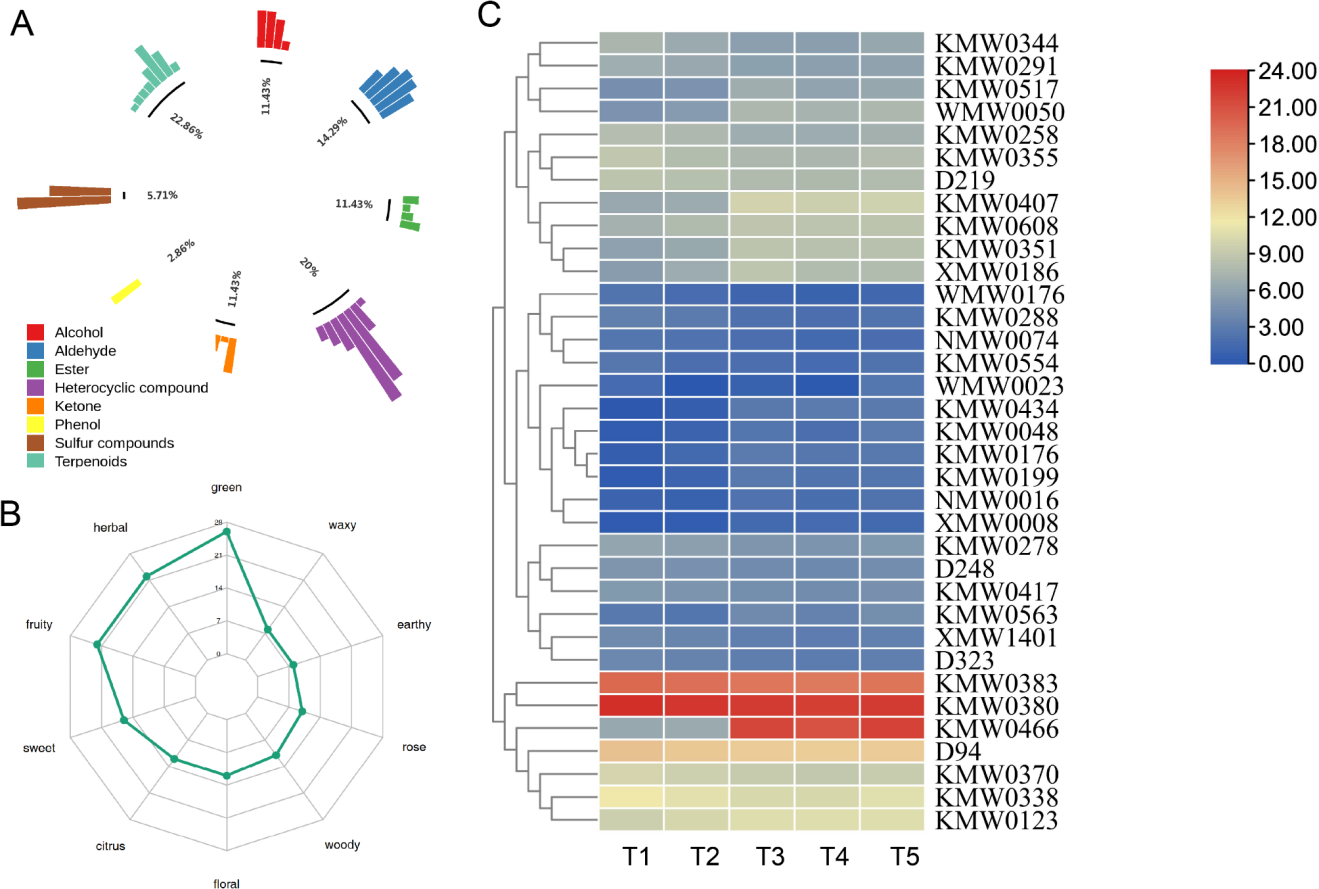
Key volatile components analysis in FTLs (**A**) Metabolite primary classification proportion circle chart; (**B**) T1 vs. T5 flavor radarchart; (**C**) The heatmap of 35 rOAVs

To further explore the effects of core volatile components on aroma formation in FTLs during the curing process, we calculated the relative odor activity values (rOAVs) of these volatiles. An rOAV ≥ 1 indicates a compound direct contribution to the flavor of the sample. Annotated information was available for 279 out of the 545 metabolites. The rOAV analysis revealed that 82 of these metabolites had OAVs > 1 at all stages, and 35 were identified as significantly differential volatiles (SDVs) based on a variable importance in projection (VIP) score ≥ 1.0, a fold change ≥ 2 or ≤ 0.5, and P-value ≤ 0.05 (Additional file 3: Table S2). Among the 35 SDVs, terpenoids compounds represented for the largest proportions, accounting for 22.86%. These include WMW0023((+)-alpha-Pinene), KMW0434(2,6,6-trimethyl-1-Cyclohexene-1-carboxaldehyde), KMW0199(.beta.-Myrcene), all of which showed an increasing trend. Additionally, monoterpenoid compounds are prone to oxidation, such as the alpha-Pinene was oxidized to alpha-Pinene-oxide. The content of aldehydes was negatively correlated with the overall sensory quality score [28], accounting for 14.29% of the SDVs (Fig. 5A). Two main trends were observed among the SDVs. The first group, consisting of 16 SDVs, increased with rising temperatures. Notabaly, KMW0466 (3-Cyclohexene-1-methanethiol, α,α,4-trimethyl-) showed substantially enrichment at T3 stages, with its concentration rising from 84 to 3,913,393.75, making it a major contributor to sulfury aromas (Fig. 5C). The second group, comprising of 19 SDVs, showed a decreasing trend (Additional file 3: Table S2). Compounds such as KMW0380 (Pyrazine, 2-methoxy-3-(2-methylpropyl)-) with an rOAVs > 4719119.12, KMW0383 (2(5 H)-Furanone, 5-ethyl-3-hydroxy-4-methyl-) with an rOAVs > 384667.18, and D94 (Benzenemethanethiol) with an rOAVs > 10404.33 had consistently high rOAVs across all five stages (Fig. 5C). Compared to T1 stage, the T5 stage was characterized by compounds with green, herbal, fruity, and sweet aromas, which, combined with citrus and floral scents, created a “fresh and sweet fragrance”profile (Fig. 5B).

### Flavor wheel (rotation) of FTLs during curing process

**Fig. 6:**
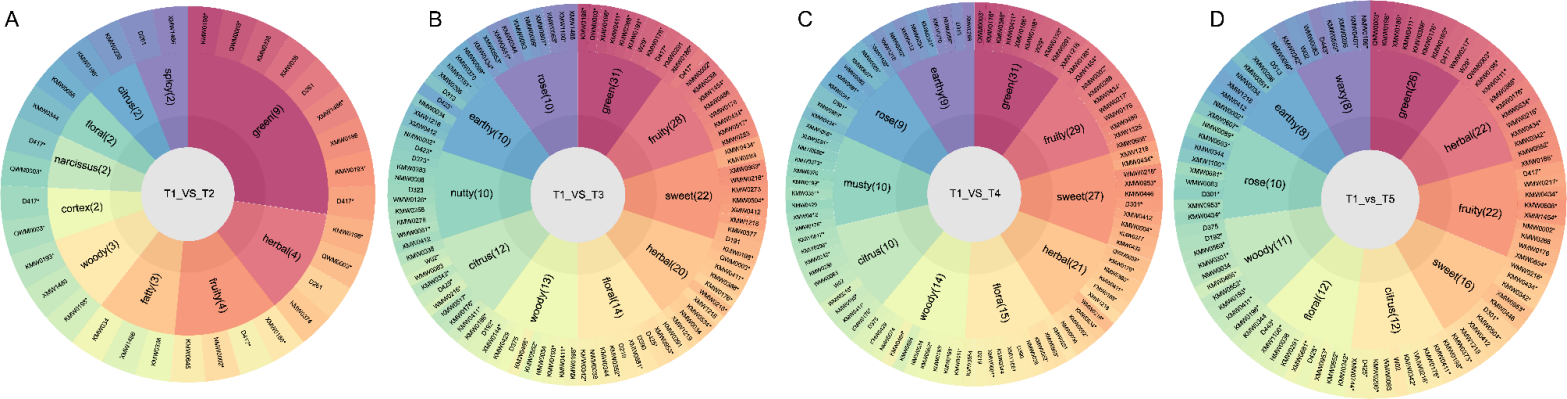
The flavor sunburst. (**A**) T1 vs. T2 (**B**) T1 vs. T3 (**C**) T1 vs. T4 (**D**) T1 vs. T5. Note: The innermost circle is the difference comparison group, the second circle is the top 10 sensory flavor characteristics with the highest number of VOCs annotated to the comparison group, the number in parentheses indicates the number of VOCs annotated to the sensory flavor characteristics, the outermost circle represents the VOCs, * represents the VOCs is up-regulated, and the rest is down-regulated. If the number of VOCs in a sensory flavor characteristic is more than 10, the top 10 differential metabolites with the largest VIP value are displayed

During the curing process, the color, aroma, and taste of tobacco underwent significant changes, with each stage of flue-curing exhibiting distinct flavor characteristics (Fig. 6). The appearance evolved from “green → yellow → brown”, while aroma transitioned from “green fragrance → fruity/herbal fragrance → ripe fruity/cortex fragrance → sweet/burnt/waxy fragrance”. As the curing progressed, the aroma of the tobacco became progressively richer, more diverse, and more complex. For a detailed overview of these changes in FTLs during curing process was shown in Fig. 2A. Compared to the T1 stages, the T2 stage was dominated by a green taste, accompanied by heightened fruity aromas such as XMW0186 (3,5-Octadien-2-one, (E, E)-) and D417 (3-Hexen-1-ol, acetate), along with narcissus aromas (Fig. 6A). Additionally, the spicy flavor D261 (Butanethioic acid, 3-methyl-, S-(1-methylpropyl) ester) and XMW1486 (5-Azulenemethanol, 1,2,3,4,5,6,7,8-octahydro-the 3,8-tetramethyl-, acetate, [3 S-(3ramethyl–) gradually diminished. By the T3 stage, the flavor profile became richer (Fig. 6B), with the emergence of fruity and sweet notes being released, along with new rose, earthy, floral, and nutty flavors, while the spicy taste decreased. In the T4 and T5 stages, the aroma profile tended to stabilize (Fig. 6C and D). Notably, the highest aroma with a strong sweet fragrance characteristic was observed in T4 stage (Fig. 6C). However, the presence of a musty odor indicated that the T4 stage (45 ℃) may be susceptible to mold, requiring careful moisture control.

### Dynamic changes in non-volatile metabolites of FTLs during curing process

**Fig. 7:**
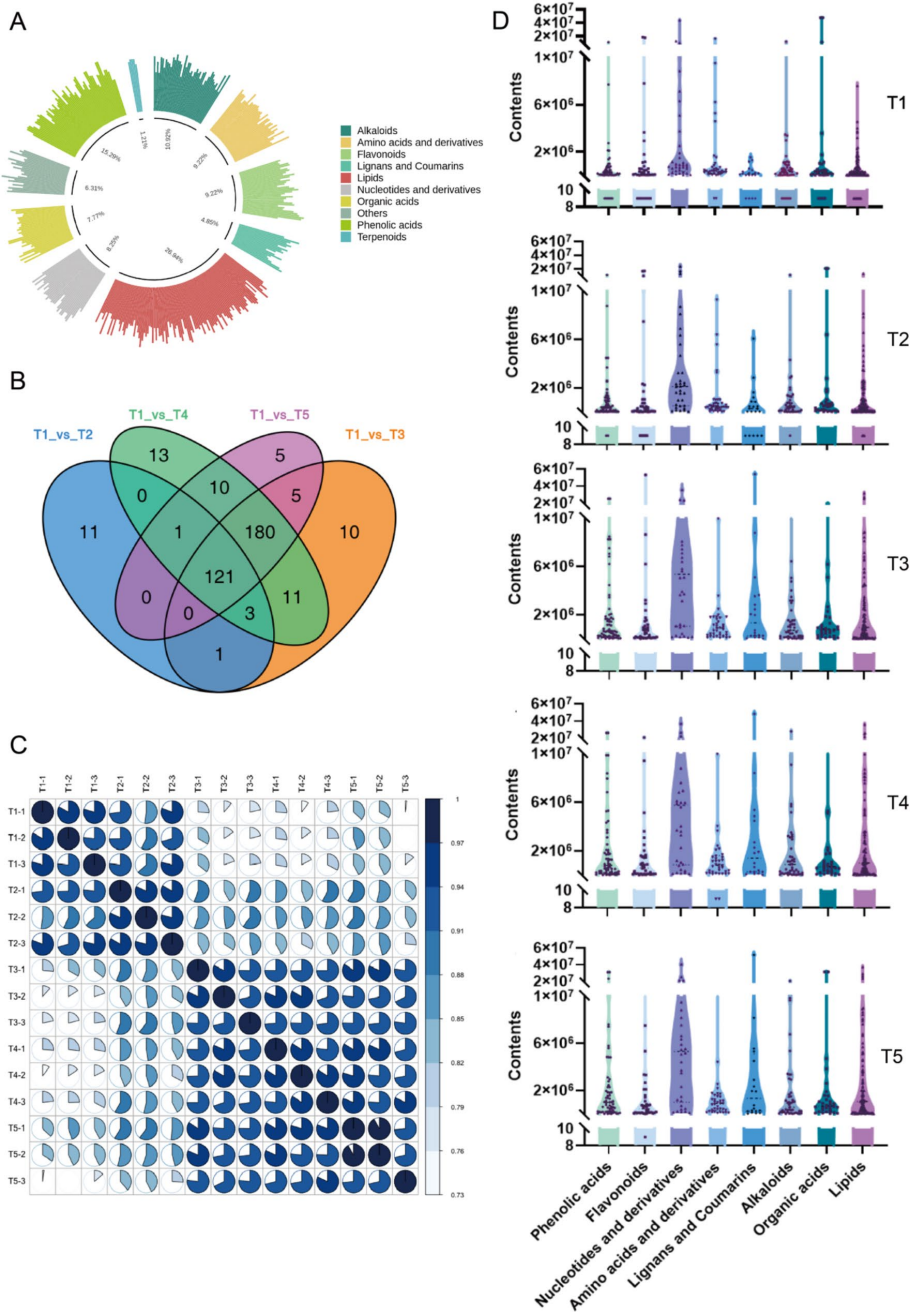
Identification of non-volatiles profiling in FTLs (**A**) Percentages of different types of non-volatiles. The outermost circle of the figure represents different types of substances and their relative contents. Each color represents a category of substances, and the length of the column generated indicates the relative content value of the substance. The innermost circle shows the ratio of the number of substances in each class to the number of substances possessed. (**B**) Venn diagram showing the numbers of common and specific differential Non-volatiles from different comparisons. (**C**) Correlation coefficient between samples. Pie chart represents pearson’s correlation coefficient of the expression of all metabolites between every two samples. (**D**) The violin diagram shows the content transformation of different substances under the temperature change of T1 ~ T5

A variety of non-volatile compounds in tobacco contribute to its rich taste profile. Using a widely targeted metabolomics approach (UPLC-MS/MS), a total of 1069 metabolites were detected, the typical total ion chromatogram (TIC) of FTLs is shown in (Additional file 4, Fig S2). A total of 412 distinct non-volatile metabolites were identified based on the threshold VIP ≥ 1, fold change ≥ 2 or fold change ≤ 0.5 (Additional file 5, Table S3). These metabolites were divided into 10 categories with a relatively balanced distribution across each category (Fig. 7A). Among them, lipids were the most prevalent, accounting for 26.94% (111 types), followed by phenolic acids (63 types, 15.2%), alkaloids (45 types, 10.92%). Flavonoids and amino acids and derivatives account the same with 38 types, dominated 9.2% (Fig. 7A). Four comparisons were conducted, including C1 (T1 vs. T2), C2 (T1 vs. T3), C3 (T1 vs. T4), C4 (T1 vs. T5)(Additional file 6, Table S4). Among them, the top 20 significant changes non-volatile metabolites in C2 comparison shows an up-grated trend. Notably, Lmmn001643 (2-Hydroxycinnamic acid*, Phenolic acids) increased with 19.58-fold, MWS20633g (O-phosphate-L-tyrosine, amino acids and derivatives) increased with 18.59-fold and Lmhn004756 (Cinnamoylferuloyltartaric acid, Phenolic acids) with 17.33-fold. All of the top 5 metabolites belongs to the categories of amino acids and derivatives and phenolic acids (Additional file 7, Fig S3). The significant up-regulation of these metabolites exerts a favorable impact on final result of FTLs. Specifically, amino acids [29], polyphenols, and alkaloids [30] are essential for the aroma quality of tobacco products [1] and are positively correlated with the total sensory score [28]. Conversely to the adorable part, flavonol glycosides, known for their contribution to the astringency of tea [31], have significantly lower astringency thresholds compared to catechins [32], making them more perceptible. Among the flavonoids, Hmcp001509 (Isorhamnetin-3-O-(2’’-O-xylosyl) glucoside-7-O-glucoside), Hmcp001628 (Limocitrin-3, 7-di-O-glucoside*), and Lmmp002463 (Sexangularetin-3-O-glucoside-7-O-rhamnoside) were predominantly found in T3 and after stages. This suggests that flavonol glycosides are a major class of compounds contributing to the bitterness of FTLs.

Venn analysis identified 121 common significant different metabolites in the four pair comparisons, indicating their ongoing role throughout the five curing stages (Fig. 7B). Additionally, C1 contained eleven, C2 had ten, C3 had thirteen, and C4 had five specific significant differential metabolites, respectively. We then evaluated and clustered the correlation of the non-volatile metabolic abundance from five stages of curing samples (Fig. 7C). The result showed a distinct separation between samples from the early stage of yellowing (T1) and the late stage of yellowing (T2) compared to other samples, while samples from at the stages of T3, T4, and T5 was more concentrated (Fig. 7C). This is also reflected in the dynamics of various classes of substances (Fig. 7D). The dynamic changes in different metabolites revealed that nucleotides and derivatives, including adenine, cytidine, and adenosine (Fig. 7D, Additional file 8, Table S5), significantly increased at the T3 stage and positively contribute to taste [33, 34]. Additionally, the content of Lignans and coumarins also increased significantly at T3 stage. Meanwhile, the content of flavonoids and alkaloids increased from T1 to T4 but gradually decreased in T5 stage.

### Combination analysis of volatile and non-volatile metabolites in KEGG

**Fig. 8:**
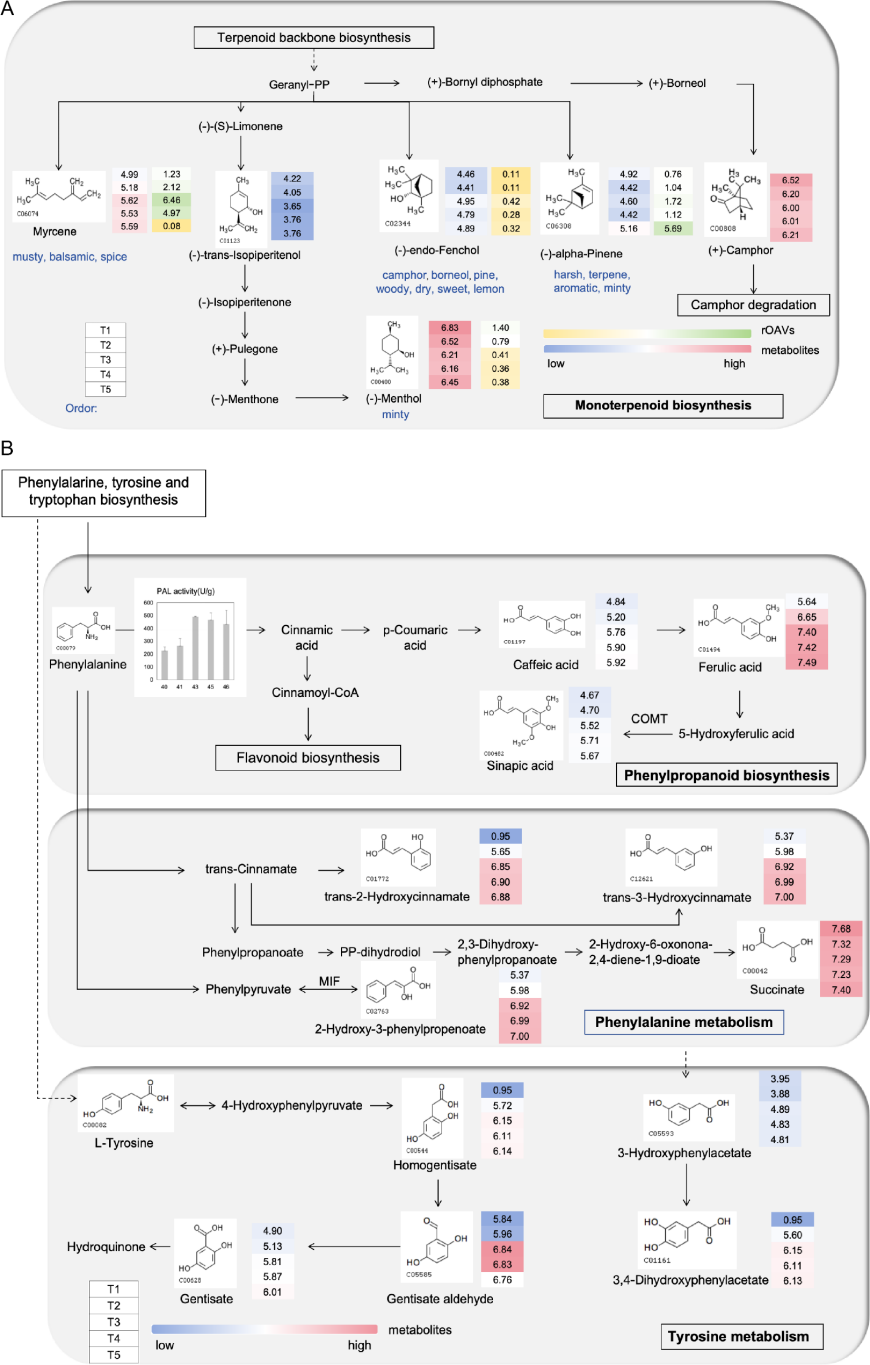
Differential analysis of secondary metabolism in FTLs.(**A**) Monoterpenoid biosynthesis (**B**) Phenylpropanoid biosynthesis, Phenylalanine and Tyrosine metabolism pathway was drawn based on KEGG database. PAL, phenylalanine ammonia-lyase [EC:4.3.1.24]; COMT, caffeic acid 3-O-methyltransferase/acetylserotonin O-methyltransferase [EC:2.1.1.68 2.1.1.4]; MIF, phenylpyruvate tautomerase [EC:5.3.2.1]

KEGG analysis of differential volatile metabolites revealed that these metabolites were significantly enriched in the monoterpenoid biosynthesis pathway (Map00902) (Fig. 8A). In this pathway, Myrcene and (-)-endo-Fenchol emitted strong musty, balsamic, spice, as well as camphor, borneol, pine, woody, dry, sweet, and lemon scents at the T3 stage. The relative odor activity value (rOAV) of (-)-Menthol was greater than 1 at the T1 stage, directly contributing to the minty odor. Subsequently, as the temperature increased, the musty, balsamic, and spice scents gradually faded. The scent of (-)-alpha-Pinene became more prominent with the increase in temperature, and its rOAV values were greater than 1 from T2 to T5. This indicates that (-)-alpha-Pinene significantly contributed to the harsh, terpene, aromatic, and minty scents during these stages.

The changes of non-volatile metabolites in the tyrosine metabolism (Map00350), phenylalanine metabolism (Map00360), and phenylpropanoid biosynthesis (Map00940) pathways were analyzed. These pathways were closely related to phenylalanine ammonia-lyase (PAL) enzyme (Fig. 8B). In the tyrosine metabolism pathway (Map00350), 3-hydroxyphenylacetate is downstream of phenylalanine metabolism and is subsequently converted into 3,4-dihydroxyphenylacetate. The contents of these two metabolites peaked at the T3 stage. In the hydroquinone synthesis pathway, the metabolite homogentisate is converted into gentisate aldehyde and gentisate. The contents of these compounds all increased to varying degrees with temperature changes (Fig. 8B). In the phenylpropanoid biosynthesis pathway (Map00940), PAL activity promotes the production of caffeic acid, which is then converted into ferulic acid and sinapic acid. The content of caffeic acid increased from 4.84 to 5.92, making a positive contribution to the sensory score [28]. In the phenylalanine metabolism pathway (Map00360), trans-2-Hydroxycinnamate, trans-3-Hydroxycinnamate, and 2-Hydroxy-3-phenylpropenoate have the same molecular formula, and their contents all increased to different extents during the flue-curing of tobacco leaves (FTLs). In this study, succinate was highly expressed from T1 to T5. As a key intermediate in the tricarboxylic acid (TCA) cycle, succinate can regulate cell metabolism and slow down cell senescence to a certain extent [35]. Under hypoxic conditions, the accumulation of succinate may be involved in regulating the expression of genes related to anaerobic respiration in plants, helping plants adapt to low-oxygen environments. Moreover, succinate is a critical mediator of the hypoxic response and is involved in protein succinylation, a newly discovered post-translational modification [36, 37].

## Discussion

The flue-curing process of tobacco leaves involves a series of complex processes, including enzyme catalysis and the transformation of intrinsic substances, ultimately achieve a desirable postharvest cigarette with softening and sweetening. During the investigation of changes in leaf phenotype, enzyme activity, and biochemical substances throughout the curing process, it was observed that the tobacco leaves underwent significant changes after baking. Specifically, when FTLs transition from the yellowing stage to the color fixing period and are removed from the roasting room, their surface color tends to darken to a brown hue. This darkening is likely due to the photooxidation-induced blackening of hydroquinone, which is an antioxidant and is photosensitive compound [38]. Polyphenol oxidase (PPO) and peroxidase (POD) enzymes, which are closely related to browning reactions [39], exhibit high activity between T3 and T5 stages (Fig. 3), indicating that this temperature range is the sensitive period for browning reactions. As shown in Fig. 2A, the browning of the leaves intensified during the T4-T5 stage. Furthermore, the activity of both POD and PPO peaks at T4 stage, which coinciding with the emergence of a musty odor in the leaves (Figs. 3 and 6C), suggesting a need for additional moisture control during this phase. Before the color-fixing stage, reducing the water content in tobacco cells can inhibit PPO and POD activity, thereby regulating the speed of browning reactions and preventing excessive browning of the leaves. Additionally, superoxide dismutase (SOD), a key enzyme in biological antioxidant systems, plays a vital role by slowing the oxidation of phenols to quinones. Since oxygen is a crucial factor in enzymatic browning reactions, controlling oxygen concentration in the oven is essential for reducing the excessive browning in tobacco leaves. Previous studies have shown that higher amino acid content contributes to the umami taste [32, 34]. In this study, the content of free amino acids increased significantly with temperature changes, suggesting that this increase may influence the rise in aroma compound abundance.

Previous study on tobacco aroma has primarily focused on the synthesis difference of aroma precursors in tobacco leaves at various growth stages [1] and the differences in flavour-precursor and volatile aroma components between waste tobacco stems [40]. Studies on the dynamic changes of biochemical substances have primarily centered on the disparities of leaves following flue-curing at different leaf ages [7] and the identification of substances resulting from tobacco browning [5]. However, there has been a lack of systematic investigations regarding the dynamic alterations of substances during tobacco curing. In this study, we systematically characterized both non-volatile and volatile compounds using LC-Q-TOF-MS and HS-SPME-GC-MS techniques, and measured the dynamic changes in various biochemical substances and enzyme activities. A total of 35 significantly different volatile compounds (SDVs) were identified (Additional file 3: Table S3). Among them, the flavor-active compounds in tobacco, including terpenoids, alcohols, esters, ketones, and heterocyclic compounds, exhibited substantial changes during flue-curing process, with a strong correlation among them. The aroma of tobacco gradually shifts from green and spicy to fruity, floral, and mellow. During the T1-T3 curing stages, the aroma volatile compounds predominantly exhibit green notes, with differential metabolites KMW0466 (3-Cyclohexene-1-methanethiol, graduallyethyl-) and D114 (Sulfur compounds, allyl methyl) increasing rapidly and showing highly sensitivity at this period of temperature (Fig. 6B, Additional file 9: Fig S4). In the T3-T4 phase, as the temperature increased, polyphenolic compounds increased significantly (Fig. 7D). These compounds not only contribute specific aromas to tobacco leaves but also trigger acidic reactions when tobacco is burned, which neutralized some of the alkalinity in tobacco products and enhance the mellowness of tobacco flavor [9]. In summary, the aroma of flue-cured tobacco should not be narrowly defined as a single woody aroma. A dynamic analysis of aroma changes during the roasting process provides a better understanding of how curing technology affects aroma substances, thereby aiding in the improvement of the roasting process.

Previous studies have demonstrated a positive correlation between the level of chlorogenic acid various attributes such as aroma quality, aroma intensity, irritation, and volatility [28]. In our study, a comprehensive KEGG analysis integrating non-volatile and volatile compounds revealed that the differential metabolites emerging during the curing process were enriched in four key pathways: monoterpenoid biosynthesis, tyrosine metabolism, phenylalanine metabolism, phenylpropanoid biosynthesis. These pathways are intimately related to the synthesis of chlorogenic acid and rutin, processes that are accompanied by the changes in PAL activity. Furthermore, a notable upregulation was observed in most metabolites involved in the hydroquinone formation pathway in the phenylalanine and tyrosine metabolic networks. This suggested that these metabolites may help neutralize free radicals and protect cells from oxidative damage during the curing process, due to their antioxidant and reducing effects. These finding is consistent with the primary groups responsible for synthesizing growth and flavor compounds active during fermentation [41]. Additionally, phenylpropanoids (ko00940), a group of plant secondary metabolites derived from phenylalanine, along with alcohols linked to acids, serve as precursors for lignin biosynthesis. The contents of caffeic acid, ferulic acid, and sinapic acid in this pathway increased with temperature, which accelerated lignin formation. This increase in lignin may explain the greater toughness of FTLs compared to fresh tobacco leaves. Moreover, these acids have diverse functions: ferulic acid is known for its anti-inflammatory, antioxidant, and antimicrobial properties [42], while sinapic acid, found in spices, citrus fruits, berries, vegetables, cereals, and oilseeds, exhibits antioxidant, anti-inflammatory, anticancer, antimutagenic, antiglycemic, neuroprotective, and antibacterial activities [43]. These substances dynamically regulate the curing process of tobacco leaves, thereby influencing the final characteristics and quality of the tobacco.

This study provides valuable insights into the dynamic changes in leaf phenotype, enzyme activity, and biochemical substances throughout the curing process within the standardized curing framework, and identifies key metabolites linked to tobacco quality and aroma. However, the factors influencing the final curing quality of tobacco require further investigation. Environmental variables such as humidity, airflow, and light exposure, as well as differences in tobacco varieties, cultivation methods, and geographical origins, can all significantly influence the metabolite and aroma compound profiles. Future research should focus on these factors to improve the generalizability of the findings. A deeper understanding of how these influencing factors correlate with the dynamic changes in tobacco quality during the curing process will enrich our knowledge of how metabolic shifts affect tobacco quality, and provide more direct evidence of their impact on the final product.

## Conclusion

This study provides a comprehensive analysis of the dynamic changes in volatile substances, metabolites, enzymes, and biochemical compounds in tobacco leaves during the flue-curing process. By integrating metabolomics with enzyme activity and biochemical analysis, we identified that 43 ℃ is a critical temperature for enzyme activity and metabolite transitions, while 45 ℃ is required strict moisture control to optimize the curing process. During the T3 stage, significant enrichment of phenolic acids, amino acids, and their derivatives was observed, indicating potential candidate biomarkers for non-volatile compounds. The aroma dynamics, significantly influenced by high rOAVs such as KMW0380, KMW0383, and KMW0466, primarily contributed to the green and woody flavor of the cured tobacco. Furthermore, the study revealed that differential volatile and non-volatile metabolites are enriched in pathways such as tyrosine metabolism, phenylalanine metabolism, phenylpropanoid biosynthesis, and flavonoid biosynthesis. These pathways are associated with phenylalanine ammonia-lyase and the synthesis of chlorogenic acid and rutin, which affect the aroma quality, intensity, irritation, and volatility of tobacco. Increased temperatures were found to enhance the contents of caffeic acid, ferulic acid, and sinapic acid, as well as PAL activity, thereby accelerating lignin formation through reactions with alcohols. Overall, this research deepens our understanding of the aroma and metabolic changes in Cuibi 1(CB-1) tobacco during curing and provides valuable insights for optimizing the curing process.

## Electronic supplementary material

Below is the link to the electronic supplementary material.

Supplementary Material 1

## Data Availability

All data generated or analyzed during this study are included in this published article [and its supplementary information files].

## Abbreviations

FTLs: Flue-cured of tobacco leaves
rOAV: Relative odor activity value
HS-SPME-GC-MS: Headspace solid phase microextraction gas chromatography-mass spectrometry
VOCs: Volatile of organic compounds
SDVs: Significant different volatiles
KEGG: Kyoto Encyclopedia of Genes and Genomes
