# Supplementary figures and images for: Multiomics provides insights into dynamic changes of aromatic profile during flue-curing process in tobacco (Nicotiana tabacum L.) leaves
Source: BMC Plant Biol. 2025 Feb 24;25:244. doi: 10.1186/s12870-025-06273-8 (PMC11849165; doi:10.1186/s12870-025-06273-8)

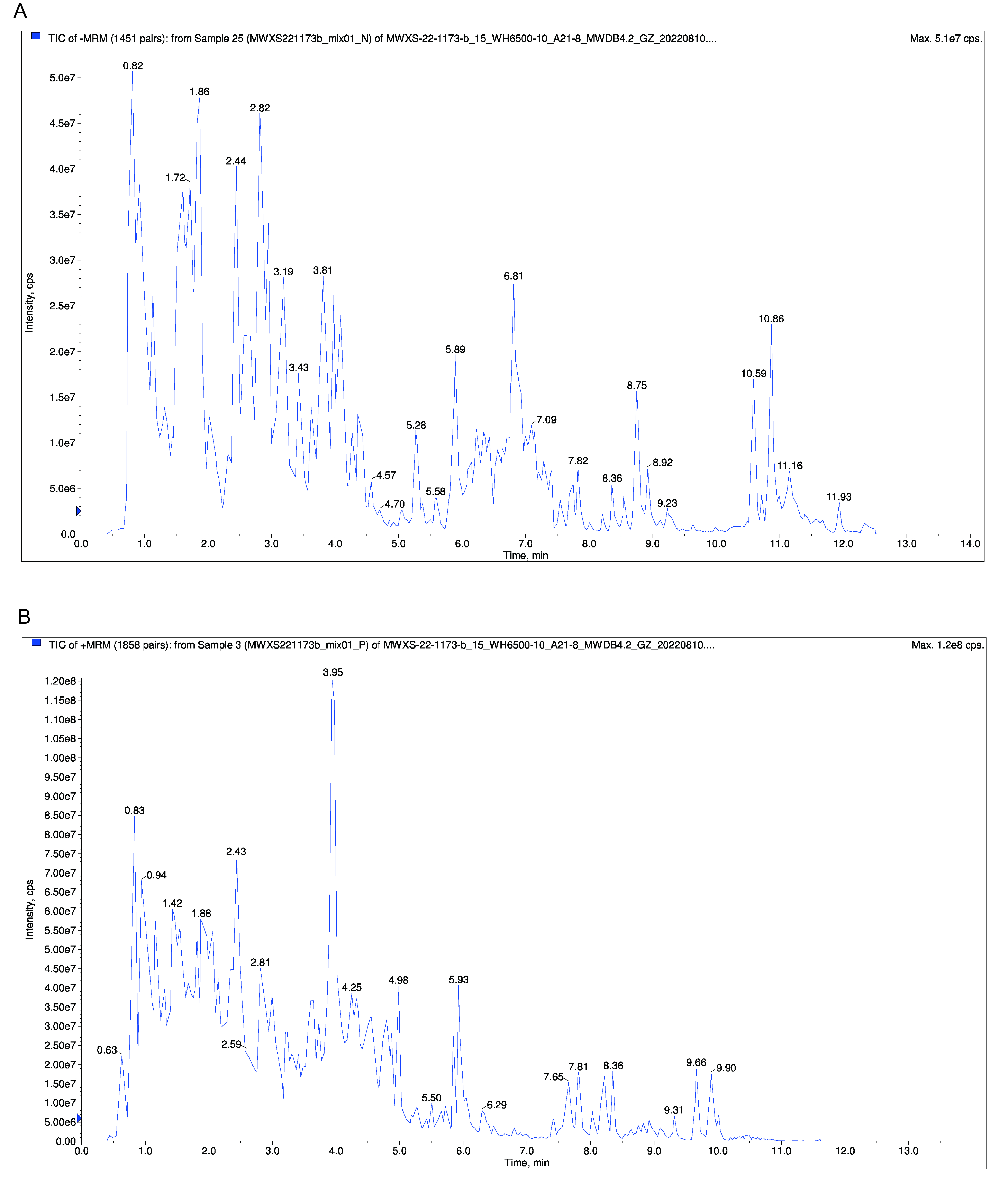

Supplement: Supplementary file 1 — Supplementary Material 1 [file 12870_2025_6273_MOESM1_ESM.zip › Additional file2/Additional file 4/Fig S2.jpg]

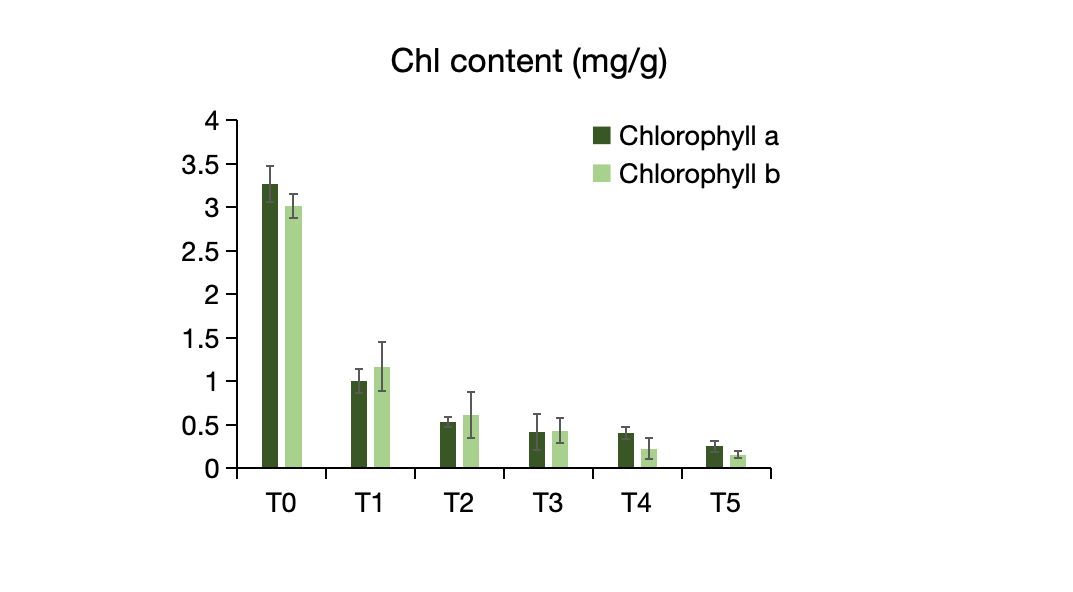

Supplement: Supplementary file 1 — Supplementary Material 1 [file 12870_2025_6273_MOESM1_ESM.zip › Additional file2/Additional file 1/Fig S1.png]

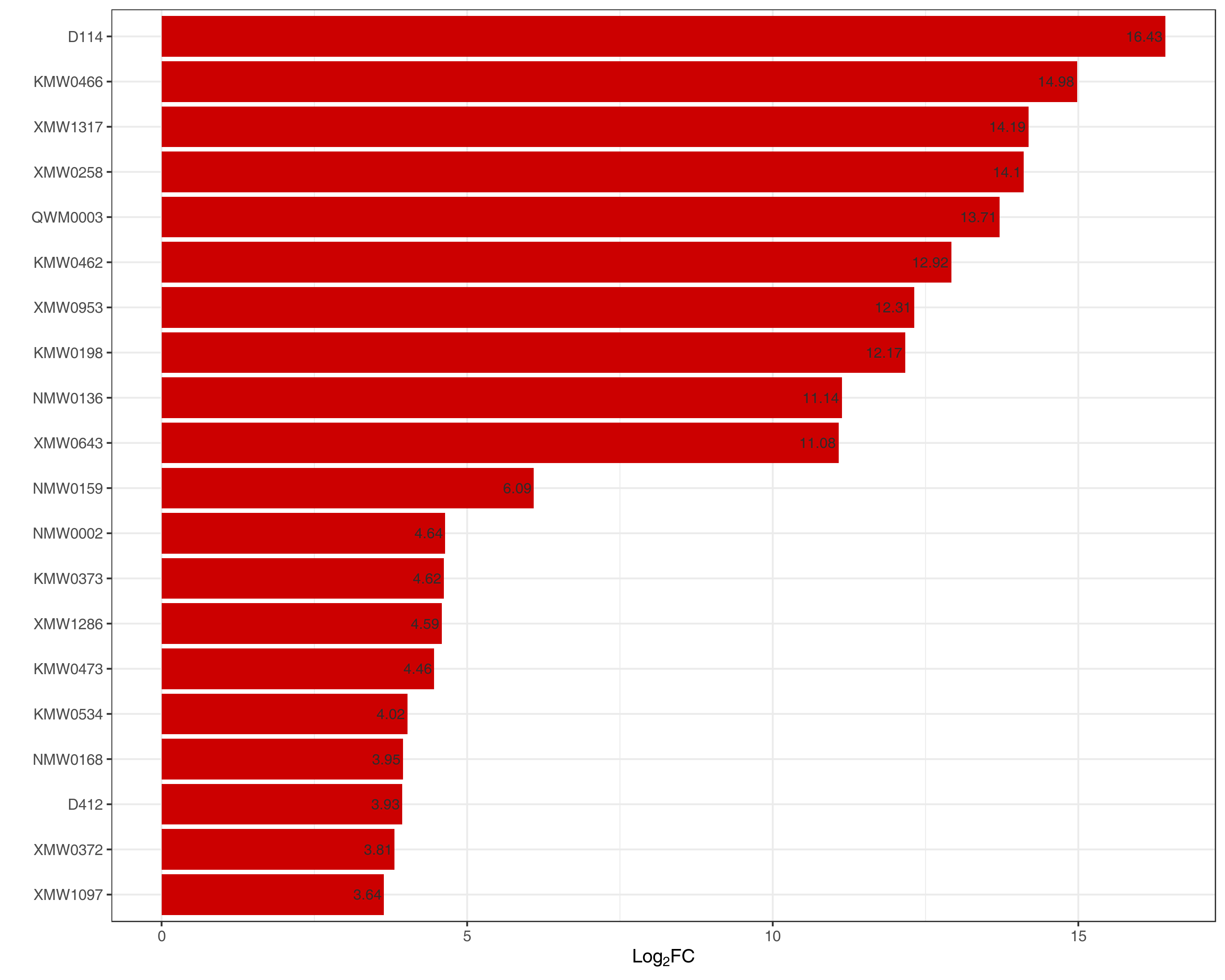

Supplement: Supplementary file 1 — Supplementary Material 1 [file 12870_2025_6273_MOESM1_ESM.zip › Additional file2/Additional file 9/Fig S4.png]

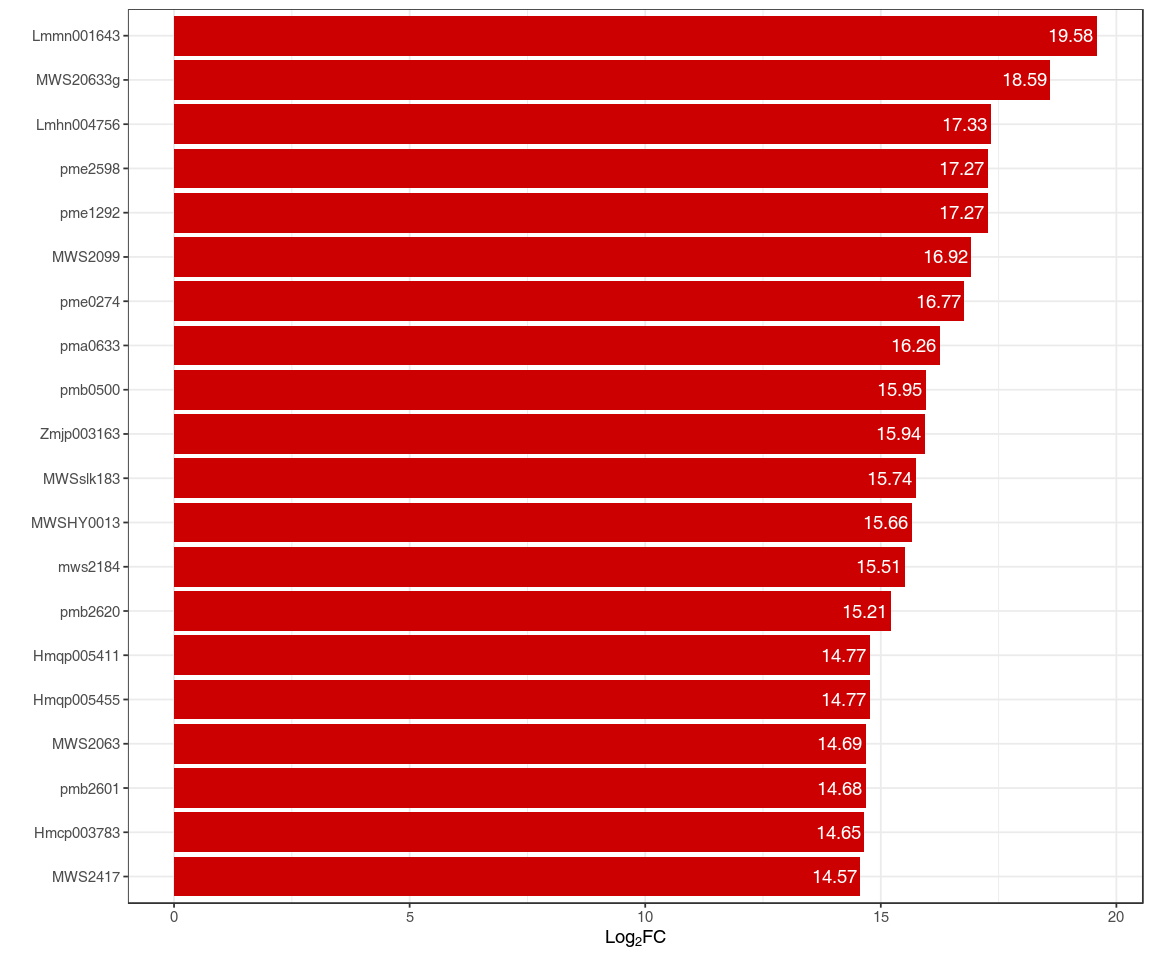

Supplement: Supplementary file 1 — Supplementary Material 1 [file 12870_2025_6273_MOESM1_ESM.zip › Additional file2/Additional file 7/Fig S3.png]
